# A viral p3a protein targets and inhibits TaDOF transcription factors to promote the expression of susceptibility genes and facilitate viral infection

**DOI:** 10.1371/journal.ppat.1012680

**Published:** 2024-11-07

**Authors:** Shuyuan Tian, Qingting Song, Yipeng Cheng, Wenmei Zhou, Kuan Wu, Yu Zhao, Yunfeng Wu, Lei Zhao

**Affiliations:** 1 State Key Laboratory for Crop Stress Resistance and High-Efficiency Production, Key Laboratory of Plant Protection Resources and Pest Management of Ministry of Education, Key Laboratory of Integrated Pest Management on Crops in Northwestern Loess Plateau of Ministry of Agriculture and Rural Affairs, College of Plant Protection, Northwest A&F University, Yangling Shaanxi, China; 2 Yangling Vocational & Technical College, Yangling Shaanxi, China; Agriculture and Agri-Food Canada, CANADA

## Abstract

The interactions among viruses and host plants are complex and fascinating because these organisms interact with and adapt to each other continuously. Many plant transcription factors play important roles in plant growth and development and in the resistance to viral infection. To facilitate the infection of plants, some viral proteins typically target and inhibit the function of plant transcription factors. In this study, we found an interesting phenomenon wherein the p3a protein of barley yellow dwarf virus (BYDV) can interact with the zinc finger domain of the TaDOF transcription factor in wheat; the zinc finger domain of TaDOF can interact with the promoter of *TaHSP70* and inhibit the transcription of the *TaHSP70* gene; and p3a interacts with the TaDOF zinc finger domain through competitive binding, alleviating TaDOF zinc finger domain-mediated inhibition of the *TaHSP70* promoter, thereby promoting *TaHSP70* expression and promoting infection by BYDV. This study demonstrates that BYDV p3a is an immunosuppressive factor and enriches our understanding of the pathogenesis of BYDV.

## Author summary

Based on past research, we conclude that some viral proteins typically target and inhibit the function of plant transcription factors to facilitate the infection of plants. In this study, we found that the p3a protein of barley yellow dwarf virus (BYDV) and the promoter of the susceptible gene *TaHSP70* can both interact with the TaDOF zinc finger domain through competitive binding. Thus, when BYDV infects plants, p3a alleviates TaDOF zinc finger domain-mediated inhibition of the *TaHSP70* promoter, thereby promoting *TaHSP70* expression and promoting infection by BYDV.

## Introduction

Over the course of evolution, plants have developed antiviral strategies such as DNA methylation, RNAi, autophagy, resistance gene expression and other resistance signaling molecule accumulation to prevent viral infection [1–3]. To infect plants, plant viruses have evolved complex mechanisms to cope with plant resistance strategies [4,5]. Plant transcription factors play important roles in antiviral processes in plants [6–8], and plant viruses have evolved strategies to combat transcription factor-induced plant immunity. Many viral proteins facilitate plant infection by targeting and inhibiting the function of plant transcription factors. For example, cassava common mosaic virus capsid proteins target the RAV1 and RAV2 transcription factors and promote viral infection by inhibiting the phosphorylation of the RAV1 and RAV2 proteins [9].

Transcription factors play important roles in plant growth and development. DOF proteins are members of a family of plant transcription factors that contain a conserved unique single zinc finger domain of Cys residues and a 50–52 amino acid DOF domain [10,11]. The DOF domain is a bifunctional domain that mediates both DNA binding and protein–protein interactions. DOF binds to the promoter of a gene and may either activate or inhibit the transcription of that gene [12]. For example, the grape DOF transcription factor VvDOF3 functions as a transcriptional activator, enhancing the transcription of genes related to resistance to powdery mildew and promoting grape resistance to powdery mildew [13]. The banana DOF transcription factor MaDOF23 acts as a suppressor and interacts with MaERF9 to regulate the expression of ripening-related genes [14]. However, there are few studies on the function of DOF transcription factors in interactions between viruses and plants. The function of the wheat TaDOF protein has also been less well studied.

In the process of virus–plant interaction, some transcription factors improve the resistance of plants to viruses by promoting the expression of downstream resistance genes. There are also several plant genes that promote viral infection. For example, *Nicotiana benthamiana* ribosomal protein large subunit 1 (NbRPL1) promotes infection by tobacco vein banding mosaic virus; *N*. *benthamiana* vicinal oxygen chelate (NbVOC1) promotes infection by beet necrotic yellow vein virus; *N*. *benthamiana* fusion protein FAD4 (NbFAD4) positively regulates infection by turnip mosaic virus [15–17]. These viruses can use these susceptibility genes to promote infection in plants. For example, the protein HSP70 has been reported to be associated with viral infections in plants [18]. Many plant viruses recruit the HSP70 protein to assist in viral replication, proper protein folding, synthesis, and assembly of virions [19]. Tomato yellow leaf curl virus (TYLCV), tomato mosaic virus (ToMV) and Chinese wheat mosaic virus (CWMV) all recruit host HSP70 proteins to complete the infection process [20–22]. Studies have also shown that beet yellows virus must interact with HSP70 to travel long distances, revealing a new way by which HSP70 promotes viral infection [23]. HSP70s are a complex multigene family, with 61 HSP70 species identified in tobacco [24]. Eighty-four HSP70 species have been identified in hexaploid wheat [25]. However, the mechanism through which TYLCV, ToMV and CWMV regulate the expression of HSP70 to promote viral infection needs further study.

Wheat yellow dwarf disease is a global cereal disease caused by infection with barley yellow dwarf virus (BYDV) and is highly harmful in the wheat-producing region of North China [26]. BYDV is a member of the family *Solemoviridae* and is spread in the field by aphids in a nonpersistent manner. Wheat plants infected with BYDV exhibit symptoms such as inverted V-shaped yellowing of leaves, dwarfing, and reduced tillering; this infection can thus severely reduce crop yield [27,28]. The BYDV genome encodes 7 proteins, among which the function of the p3a protein encoded by BYDV during viral infection is still unclear. Previous studies in our laboratory have shown that p3a inhibits *BAX*-induced necrosis, so it is speculated that p3a may function in inhibiting host immunity and promoting BYDV infection.

To clarify the function of the p3a protein in the process of BYDV infection in wheat, the interaction between p3a and the DOF protein in the zinc finger structural region was verified by yeast two-hybrid (Y2H) system, bimolecular fluorescence complementation (BiFC), coimmunoprecipitation (co-IP) and other assays. The zinc finger structural region of the TaDOF protein can bind to the promoter of the *TaHSP70* gene and inhibit its transcription. p3a alleviates the inhibition of the TaDOF zinc finger structure on the *TaHSP70* promoter through competitive binding. Overexpression of the TaHSP70 protein was found to promote BYDV infection. This study demonstrated that BYDV p3a is an immunosuppressive factor, laying a foundation for revealing the pathogenesis of BYDV.

## Results

### p3a inhibits plant immunity and promotes BYDV infection

**Fig 1:**
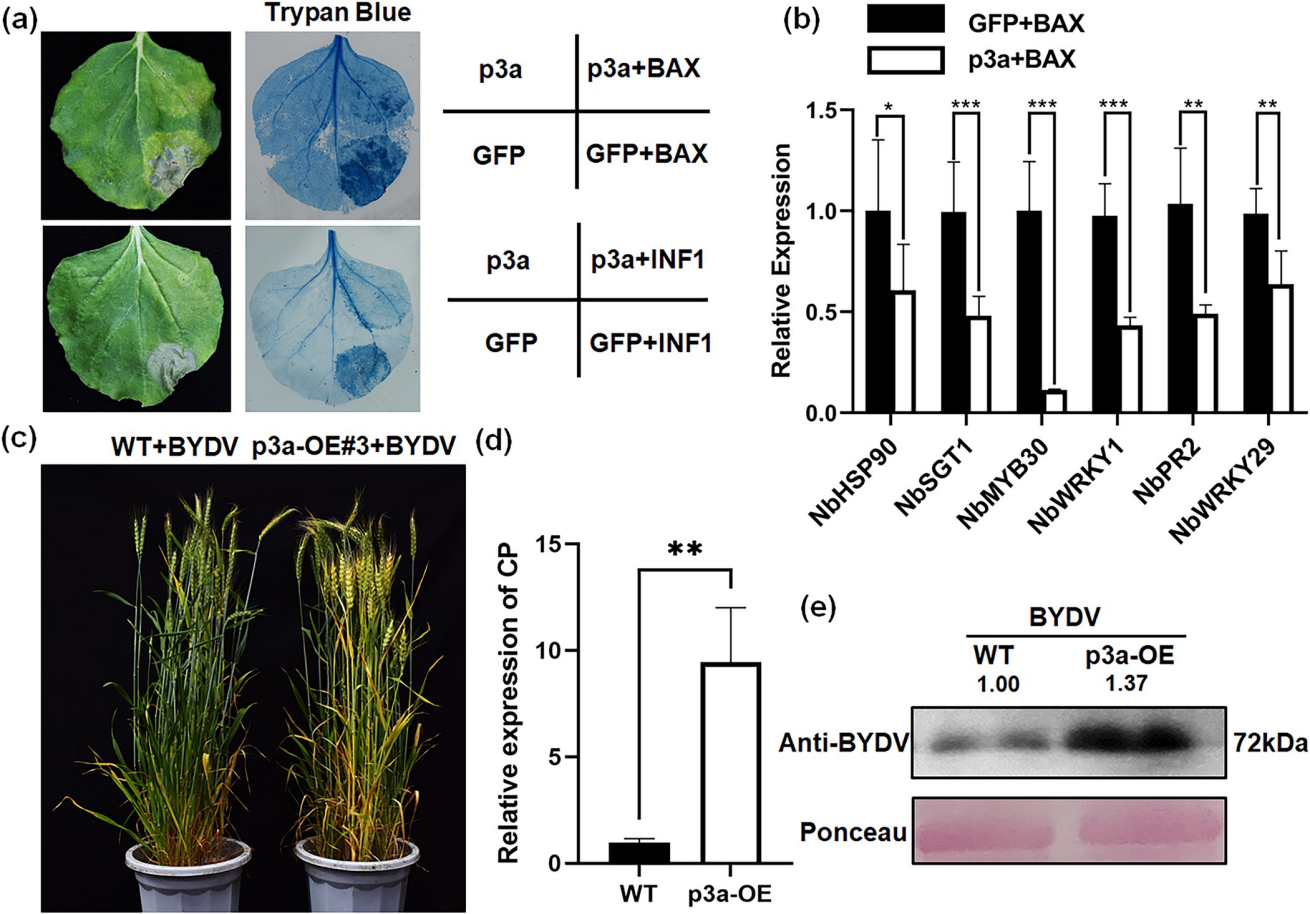
Overexpression of p3a inhibited plant immunity and promoted the accumulation of BYDV in wheat. (**a**) Overexpression of p3a in *N*. *benthamiana* inhibited BAX*-* and INF1-induced tobacco cell necrosis. Cell death was analyzed via trypan blue staining. (**b**) Overexpression of p3a in *N*. *benthamiana* inhibited the expression of immune-related genes stimulated by *BAX*. RT–qPCR was performed to measure gene expression. The values are the means ± SDs (n = 3). *P < 0.05, **P < 0.01, ***P < 0.001 (Student’s *t* test). (**c**) Phenotypes of wild-type (WT) and transgenic ‘Fielder’ plants overexpressing BYDV *p3a* (p3a-OE) and infected with BYDV. (**d-e**) Overexpression of p3a increases BYDV accumulation. The accumulation of BYDV was analyzed by RT–qPCR (**d**) and western blotting (**e**). The values are the means ± SDs (n = 3). The data were analyzed using IBM SPSS Statistics 29.0. The numbers indicate the relative gray value ratio of the BYDV band to the actin band calculated with ImageJ. *P < 0.5 (Student’s *t* test).

Seven proteins encoded by BYDV were transiently coexpressed in *N*. *benthamiana* with *BAX* and *INF1*. *BAX* is a mouse gene, and *INF1* is a gene from *Phytophthora parasitica*. Both of these genes can stimulate cell necrosis in *N*. *benthamiana*, and they are often used to screen for proteins that inhibit the immune response in plants [29–32]. BYDV p3a inhibited cell necrosis caused by *INF1* in addition to *BAX* (Fig 1A). In addition, the results of quantitative reverse transcription polymerase chain reaction (RT-qPCR) showed that p3a inhibited the expression of the *BAX*-stimulated immune-related genes *NbHSP90*, *NbSGT1*, and *NbMYB30* and other genes (Fig 1B).

To further dissect the effects of p3a on wheat development and BYDV infection, we generated transgenic ‘Fielder’ lines overexpressing *p3a* (*p3a*-OE; Figs 1C and S1A–S1C). The *p3a* transgenic ‘Fielder’ lines did not appear to be significantly different from the wild-type (WT) ‘Fielder’ line. However, after BYDV inoculation, the WT wheat plants exhibited the typical symptoms of leaf tip yellowing; the leaf tip yellowing symptoms of *p3a*-OE plants intensified, and the number of leaves that exhibited yellowing also increased. RT-qPCR (Fig 1D) and western blotting (Fig 1E) assays also revealed that p3a overexpression promoted BYDV infection.

### p3a interacts with the zinc finger structure region of TaDOF

**Fig 2:**
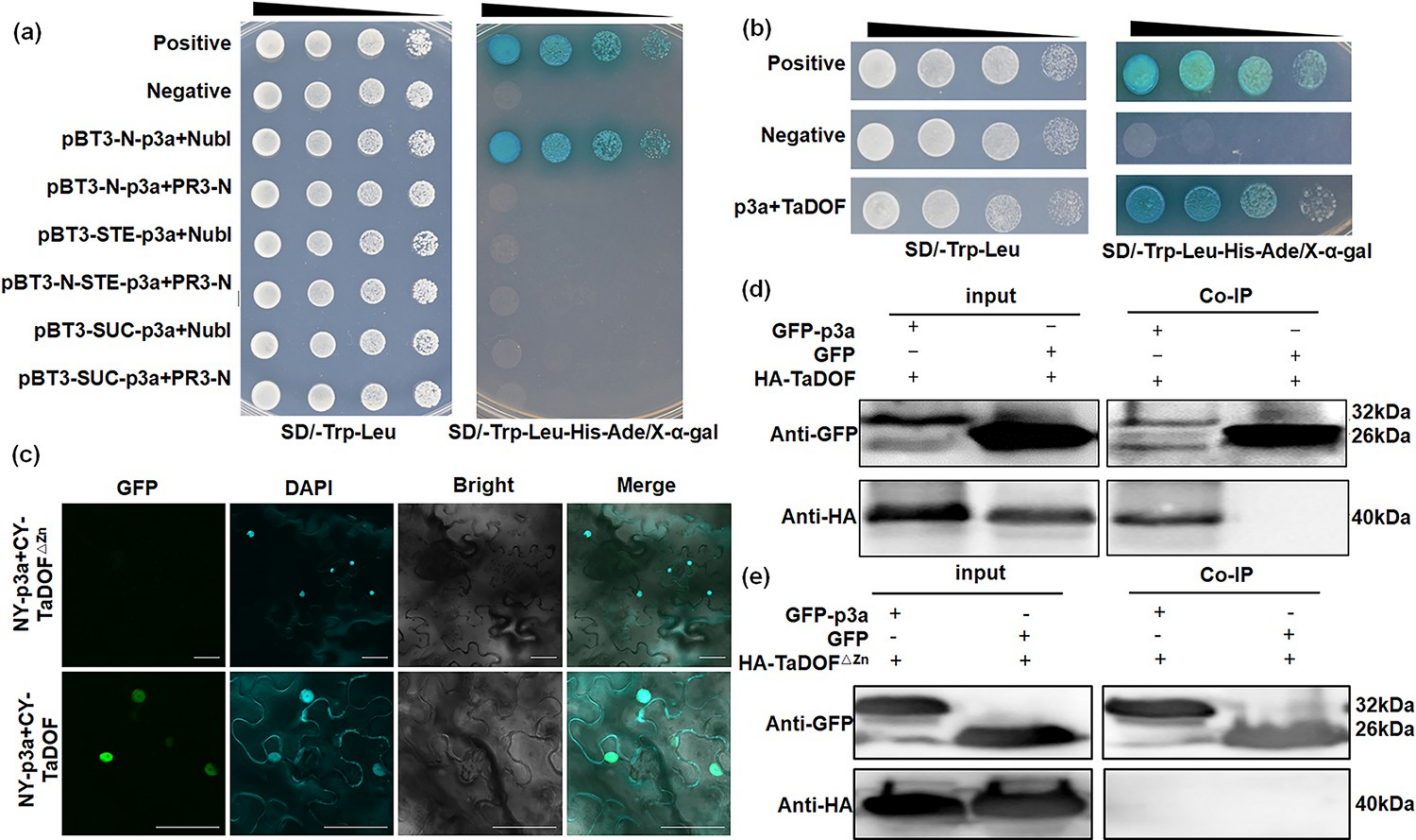
p3a interacts with the zinc finger domain of the transcription factor TaDOF in the nucleus. (**a**) Screening the optimal vector for the yeast two-hybrid (Y2H) assay. (**b**) The interaction between p3a and TaDOF was verified by Y2H. pNubG-Fe65+pTSU2-APP was the positive control, and pNubG-Fe65+pPR3-N was the negative control. (**c**) Bimolecular fluorescence complementation (BiFC) confirmed that p3a and TaDOF interact in the nucleus and that they no longer interact in the absence of the zinc finger domain of TaDOF. The nuclear localization was determined with DAPI. (**d-e**) Coimmunoprecipitation (co-IP) verified that p3a and TaDOF interact *in vivo* (**d**) but no longer interact in the absence of the zinc finger domain of TaDOF (**e**).

As predicted by TMHMM online software (https://dtu.biolib.com/DeepTMHMM), p3a has a transmembrane structure, and its transmembrane segment is located at amino acids 6–31 (S2A Fig). Subcellular localization analysis revealed that p3a can be targeted to multiple subcellular compartments, including the nucleus, in *N*. *benthamiana* cells (S2B Fig). Therefore, the protein that interacts with p3a was screened by the split-ubiquitin Y2H system. p3a was inserted into different Y2H screening library vectors for Y2H experiments, and the pBT3-N-p3a vector was found to be suitable for use in the p3a library screening assay (Fig 2A). The Y2H library screening assay identified 14 proteins that might interact with p3a (S1 Table). After further verification by Y2H (Fig 2B), BiFC (Fig 2C) and co-IP (Fig 2D) assays, we found that p3a interacts with TaDOF. Moreover, TaDOF is a transcription factor that interacts with p3a in the nucleus. Domain analysis of TaDOF revealed that TaDOF contains a zinc finger structure (S2C Fig). The co-IP assay showed that p3a could no longer interact with TaDOF after the zinc finger region was removed from TaDOF (Fig 2E).

### p3a can inhibit TaDOF-mediated resistance of wheat to BYDV

**Fig 3:**
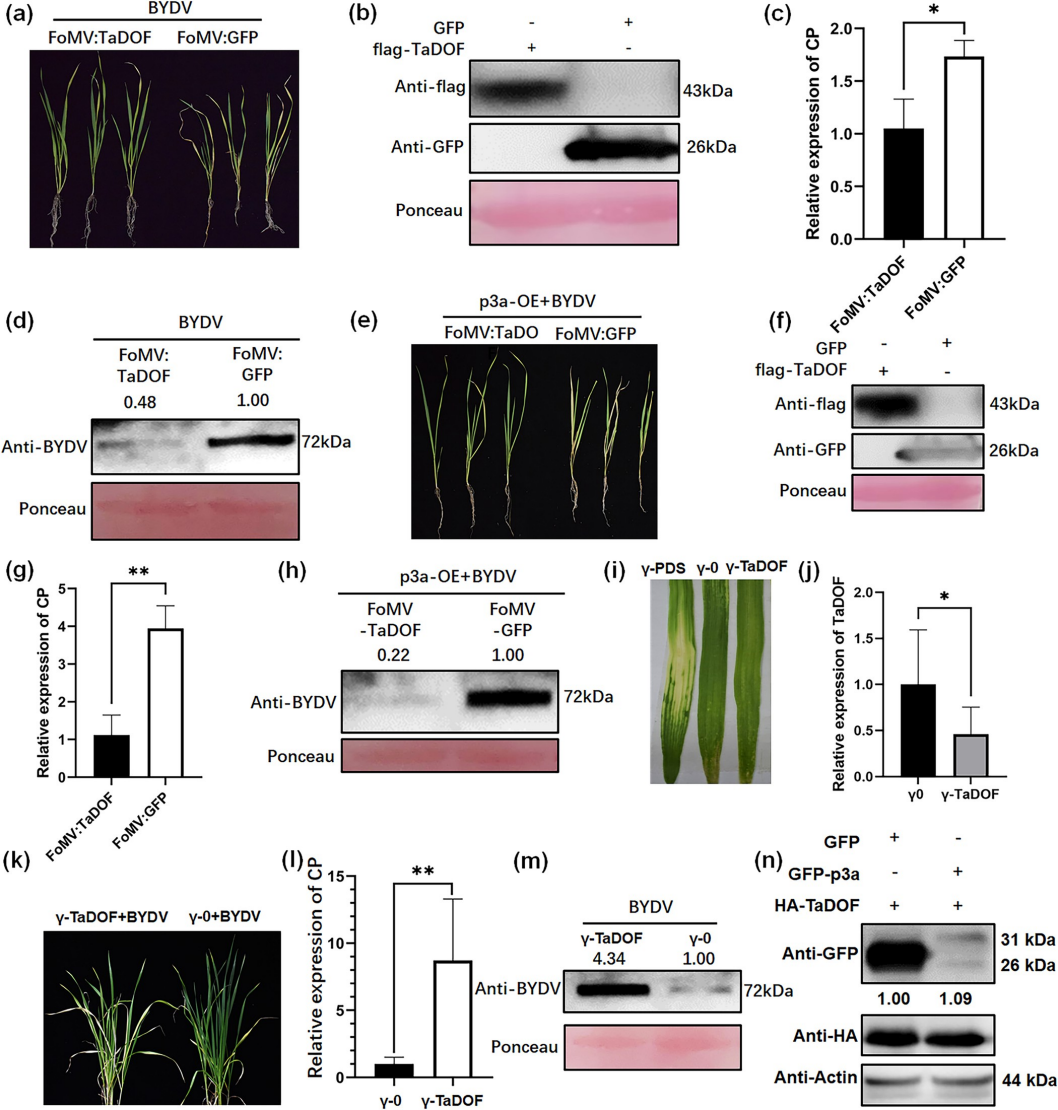
Function of TaDOF in BYDV infection in wheat. (**a-d**) BYDV infection was inhibited by *TaDOF* overexpression mediated by the PV101 vector in ‘Fielder’ wheat (**a**). Western blot analysis showed TaDOF expression in wheat (**b**). BYDV *CP* transcript levels and BYDV levels were analyzed by RT–qPCR (**c**) and western blotting (**d**). The values are the means ± SDs (*n* = 3). *P < 0.05 (Student’s *t* test). (**e-h**) p3a pathogenicity was weakened by PV101-mediated *TaDOF* overexpression in p3a transgenic ‘Fielder’ wheat (**e**). Western blot analysis showed TaDOF expression in wheat (**f**). The BYDV *CP* transcript levels and BYDV levels were analyzed by RT–qPCR (**g**) and western blotting (**h**). The values are the means ± SDs (*n* = 3). **P < 0.01 (Student’s *t* test). (**i-m**) Photobleaching mediated by BSMV (**i**) and the silencing efficiency of TaDOF (**j**). γ0 was used as the negative control, and γ-PDS was used as the positive control. Silencing *TaDOF* promoted the formation of wheat yellowing symptoms caused by BYDV (**k**). BYDV *CP* transcript levels and BYDV levels were analyzed by RT–qPCR (**l**) and western blotting (**m**) after *TaDOF* silencing. The values are the means ± SDs (*n* = 3). The RT–qPCR data were analyzed using IBM SPSS Statistics 29.0. The numbers indicate the relative gray value ratio of the BYDV band to the actin band calculated with ImageJ. **P < 0.01 and *P < 0.05 (Student’s *t* test). (**n**) Overexpression of *GFP-p3a* did not affect the protein content of 3HA-TaDOF. The coexpression of *GFP* and 3*HA-TaDOF* was used as a negative control. The numbers indicate the relative gray value ratio of the HA band to the actin band, as calculated with ImageJ.

*TaDOF* overexpression mediated by the PV101 vector in ‘Fielder’ wheat plants inhibited BYDV infection (Fig 3A–3D). Compared with the control group, *TaDOF*-overexpressing wheat plants showed reduced yellowing and dwarfing symptoms of BYDV infection. The RT–qPCR and western blotting results also showed that the BYDV content decreased significantly after *TaDOF* overexpression. Overexpression of *TaDOF* in *p3a*-OE ‘Fielder’ wheat plants also significantly inhibited BYDV infection (Fig 3E–3H). Similarly, *TaDOF*-overexpressing wheat plants showed reduced yellowing and dwarfing symptoms of BYDV infection relative to the controls. In contrast, silencing *TaDOF* in wheat significantly promoted BYDV infection (Fig 3I–3M). Approximately 14 days after virus-induced gene silencing (VIGS) of *TaDOF*, photobleaching occurred in the positive control wheat plants in which the *TaPDS* gene was silenced (Fig 3I). RT-qPCR analysis showed that the *TaDOF* gene had also been successfully silenced, with a silencing efficiency of 55% (Fig 3J). Then, BYDV was inoculated, and 7 days later, BYDV content was analyzed by RT-qPCR (Fig 3L) and western blotting (Fig 3M). The results showed that silencing TaDOF significantly promoted symptom formation and viral accumulation in BYDV (Fig 3K–3M). Western blotting analysis revealed that overexpression of p3a had no significant effect on the protein content of TaDOF (Fig 3N).

### p3a promotes the expression of *TaHSP70* by alleviating the inhibitory effect of TaDOF on the downstream gene *TaHSP70*

**Fig 4:**
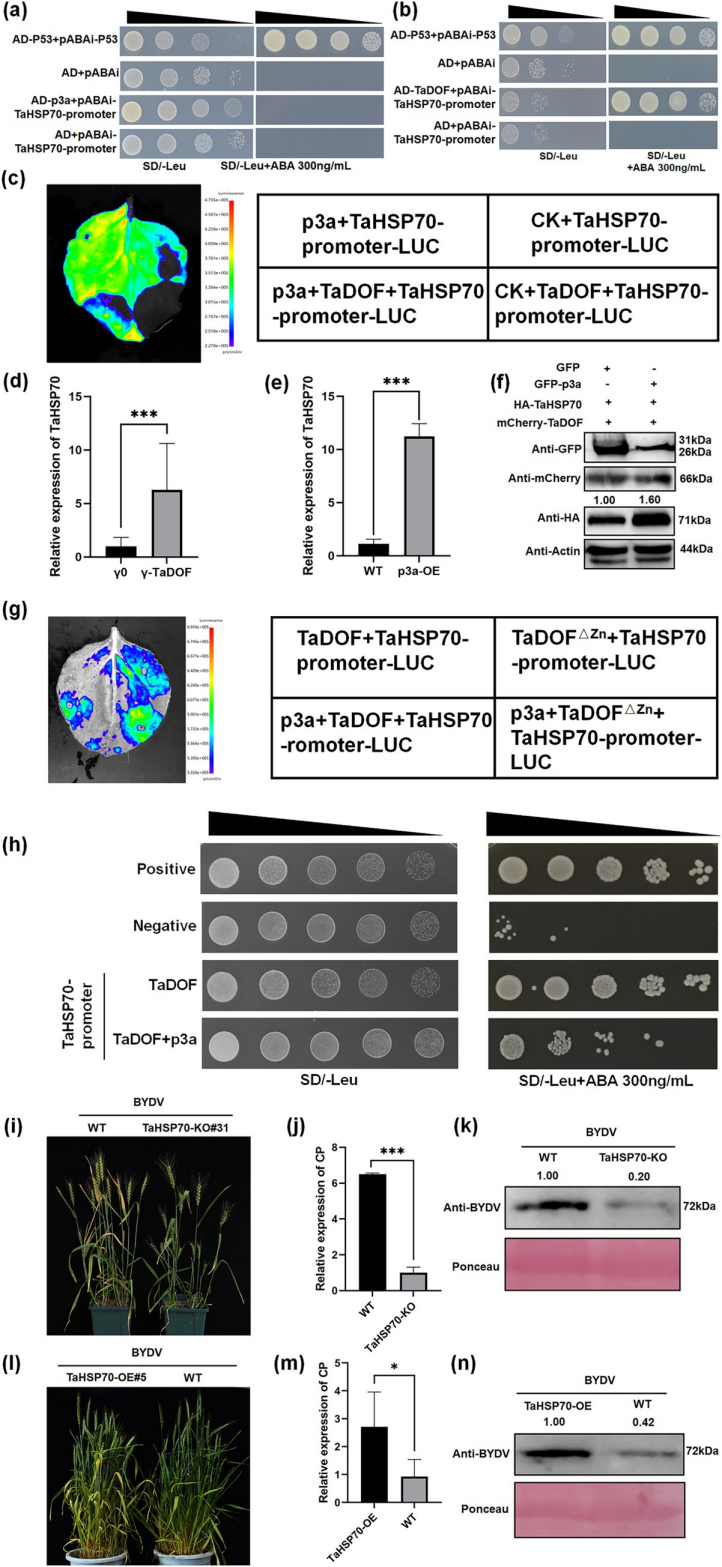
p3a alleviates the transcriptional inhibition of TaHSP70 by TaDOF and promotes infection by BYDV. (**a-b**) A yeast one-hybrid (Y1H) assay confirmed that TaDOF interacts with the promoter of *TaHSP70* (**a**), while p3a does not interact with the promoter of *TaHSP70* (**b**). (**c**) Dual-luciferase analysis showed that overexpression of p3a promoted the expression of *TaHSP70* promoter-activated LUC, while TaDOF inhibited the expression of *TaHSP70* promoter-activated LUC. Overexpression of *p3a* partially eliminated the inhibitory effect of TaDOF on the *TaHSP70* promoter. (**d**) After *TaDOF* silencing, *TaHSP70* gene expression was promoted, as determined by RT–qPCR. The values are the means ± SDs (*n* = 3). (**e**) The expression of TaHSP70 was quantitated in wild-type (WT) and *p3a*-OE wheat. WT was used as the negative control. The values are the means ± SDs (n = 3). **P < 0.01 (Student’s *t* test). (**f**) TaDOF, 3*HA*-*TaHSP70* and *GFP-p3a* were coinfiltrated and expressed simultaneously in *N*. *benthamiana*, and *GFP-p3a* promoted the protein content of 3*HA-TaHSP70*. The coexpression of *GFP* and 3*HA-TaHSP70* was used as a negative control. The numbers indicate the relative gray value ratio of the HA band to the actin band, as calculated with ImageJ. (**g**) Dual-luciferase analysis showed that after deletion of the *TaDOF* zinc finger domain, *TaDOF-*mediated inhibition of *TaHSP70* promoter activity was reduced. (**h**) Y1H staining showed that *p3a* expression inhibited the interaction of TaDOF with the *TaHSP70* promoter. AD-P53+pAbAi-P53 was used as the positive control, and AD+pAbAi was the negative control. (**i**) Knockout of *TaHSP70* inhibited the formation of wheat yellowing symptoms caused by BYDV. (**j-k**) The BYDV *CP* transcript levels and BYDV levels were analyzed by RT–qPCR (**j**) and western blotting (**k**). (**l**) Overexpression of *TaHSP70* inhibits the formation of wheat yellowing symptoms caused by BYDV. (**m-n**) The BYDV *CP* transcript levels and BYDV levels were analyzed by RT–qPCR (**m**) and western blotting (**n**). The values are the means ± SDs (*n* = 3). The numbers indicate the relative gray value ratio of the BYDV band to the actin band calculated with ImageJ. *P < 0.05, ***P < 0.001 (Student’s *t* test).

After screening the aureobasidin A (AbA) concentration, 300 ng/mL AbA was found to completely inhibit the self-activation of the pAbAi-TaHSP70 promoter (S3 Fig). Yeast one-hybrid (Y1H) assays showed that TaDOF could interact with the promoter of *TaHSP70*, while p3a could not interact with the promoter of *TaHSP70* directly (Fig 4A and 4B). The *TaHSP70* promoter was used to activate the expression of LUC, and the addition of p3a promoted the activity of the *TaHSP70* promoter, while the addition of TaDOF inhibited the activity of the *TaHSP70* promoter (Fig 4C). The expression of *TaHSP70* in ‘Fielder’ wheat plants significantly increased after *TaDOF* was silenced (Fig 4D), and the overexpression of *p3a* in ‘Fielder’ wheat plants promoted the expression of *TaHSP70* (Fig 4E). Overexpression of *p3a* in *N*. *benthamiana* also significantly promoted the protein content of TaHSP70 (Fig 4F). These findings indicate that TaDOF may inhibit the transcription of *TaHSP70*, while p3a may activate the transcription of *TaHSP70*.

Moreover, the zinc finger domain of TaDOF is a key domain for the interaction between p3a and TaDOF (Fig 2C and 2E). Therefore, we speculated that p3a inhibited the interaction between TaDOF and the promoter of *TaHSP70* by competitively binding the zinc finger domain of TaDOF. When the zinc finger domain of TaDOF was removed, the inhibitory effect of TaDOF on the *TaHSP70* promoter was weakened (Fig 4G), indicating that the zinc finger domain was the main domain via which TaDOF inhibited *TaHSP70* promoter activity. The interaction between TaDOF and the *TaHSP70* promoter was also weakened in the presence of p3a (Fig 4H).

After BYDV inoculation of *TaHSP70*-knockout ‘CB037’ wheat plants, the number of yellowed leaves and the severity of yellowing were significantly reduced compared with the control group (Figs 4I and S4). This result suggests that *TaHSP70* knockout inhibited BYDV infection. This finding was also confirmed by RT–qPCR and western blotting analysis of the BYDV content (Fig 4J and 4K). Moreover, we found that *TaHSP70*-knockout mutants exhibited reduced plant height compared with WT (Figs 4I and S4). After BYDV inoculation in ‘Fielder’ wheat plants overexpressing *TaHSP70*, the number of yellowed leaves and the severity of yellowing were significantly increased compared with the control group (Figs 4L and S5). This finding further suggests that *TaHSP70* can promote BYDV infection, which was also confirmed by RT–qPCR and western blotting analysis of the BYDV content (Fig 4M and 4N). These results indicated that p3a promoted the expression of *TaHSP70* by inhibiting the interaction between TaDOF and the promoter of *TaHSP70* and ultimately promoted BYDV infection.

### Both the transmembrane domain and the 33rd amino acid of p3a are key regions for the interaction of p3a with TaDOF and the promotion of *TaHSP70* expression

**Fig 5:**
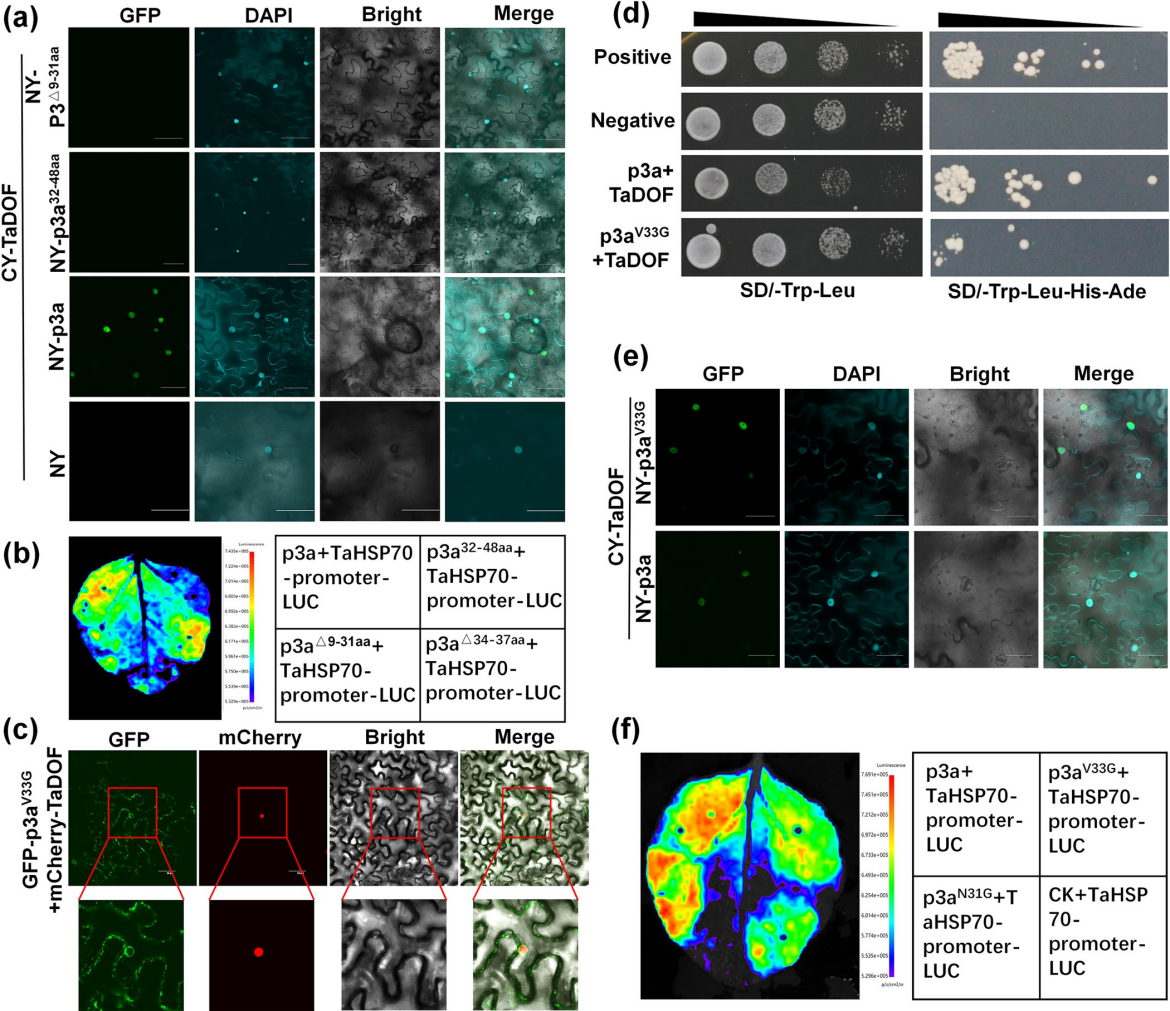
Both the transmembrane domain and the 33rd amino acid of p3a are key regions for the interaction of p3a with TaDOF and the promotion of *TaHSP70* expression. (**a**) BiFC assays showing the interaction between p3a (and its mutants) and TaDOF. Leaves of *N*. *benthamiana* were agroinfiltrated with a mixture of *A*. *tumefaciens* strains expressing the NYFP-p3a (or p3a mutant) or CYFP-TaDOF construct. YFP signals were observed at 3 dpi. Infiltration with *Agrobacterium* expressing the p3a construct alone was used as the negative control. (**b**) Dual-luciferase analysis showed that when amino acids 1–30 of p3a are absent, amino acids 31–47 of p3a alone can no longer promote *TaHSP70* promoter-activated LUC. When p3a is absent from the transmembrane domain (amino acids 7–31), overexpression of *p3a* (amino acids 1–7 and 31–47) can no longer promote *TaHSP70* promoter-activated LUC. Deletion of amino acids 34–37 by p3a did not affect the transcriptional activation of the *TaHSP70* promoter. (**c**) After the 33rd amino acid of p3a was mutated, the localization of p3a to TaDOF in the nucleus was reduced. Leaves of *N*. *benthamiana* were agroinfiltrated with a mixture of *A*. *tumefaciens* strains expressing *GFP-p3a*^33G^ and *mCherry-TaDOF*. GFP and mCherry signals were observed at 3 dpi. (**d**) Yeast two-hybrid assays verified that the interaction between p3a and TaDOF was weakened when the 33rd amino acid of p3a was mutated. pBT3-N-p3a+pOst1-NubI was used as a positive control, and pBT3-N-p3a+pPR3-N was used as a negative control. (**e**) BiFC assays showing the interaction between p3a (and the p3a^33G^ mutant) and TaDOF. Leaves of *N*. *benthamiana* were agroinfiltrated with a mixture of *A*. *tumefaciens* strains expressing the NYFP-*p3a* (or *p3a*^*33G*^) and CYFP-*TaDOF* constructs. YFP signals were observed at 3 dpi. Infiltration with *Agrobacterium* expressing the p3a construct alone was used as the negative control. Bar, 50 μm. (**f**) Dual-luciferase analysis showed that when the 33rd amino acid of p3a was mutated, the ability of p3a to promote *TaHSP70* promoter-activated LUC was weakened. However, when the 31st amino acid of p3a was mutated, the ability of p3a to promote *TaHSP70* promoter-activated LUC was not significantly changed.

When the transmembrane region of p3a (9–31 amino acids) was removed, p3a no longer interacted with TaDOF (Fig 5A), and activation of the *TaHSP70* promoter by p3a was also abolished (Fig 5B). Numerous different amino acid deletion mutants of p3a were constructed, and these mutants were subjected to coexpression and colocalization analysis with TaDOF (S6 Fig). The results showed that when the 33rd amino acid of p3a was mutated, the colocalization of the p3a mutants with TaDOF was altered (Fig 5C), and the interaction between p3a and TaDOF was weakened (Fig 5D); however, the p3a mutant p3a^33G^ could still interact with TaDOF in the nucleus (Fig 5E). The ability of the p3a mutant to activate the *TaHSP70* promoter was also significantly reduced (Fig 5F). These results indicated that both the transmembrane domain and the 33rd amino acid of p3a play important roles in its activation of the *TaHSP70* promoter.

## Discussion

The interactions among viruses and host plants are complex and fascinating because they interact and adapt to each other continuously [33,34]. The results of this study indicate that the p3a protein of BYDV promotes the expression of *TaHSP70* by targeting and inhibiting the function of TaDOF transcription factors, thus promoting BYDV infection.

p3a was originally discovered by Smirnova et al. [35] in *Poleroviruses* and *Luteoviruses*. p3a is a small non-AUG codon initiation protein translated from the transcript of sgRNA1. p3a plays an important role in the long-distance movement of turnip yellows virus and brassica yellows virus in plants [35–37]. Deblasio et al. [38] also found that p3a forms protein complexes with the movement protein of potato leafroll virus (PLRV) in plants and collaboratively promotes the movement of PLRV. Recently, Liu et al. [39] screened p3a-interacting proteins in *Arabidopsis thaliana* and found 138 interacting proteins, including functional proteins related to the photosynthetic pathway and signal transduction pathway. However, the function of p3a in the interaction between BYDV and wheat remains unclear. In the present study, BYDV p3a was overexpressed in wheat, and it was found that BYDV p3a promoted BYDV infection. The Y2H results showed that BYDV p3a might interact with 14 wheat proteins. Through further analysis, 9 proteins were found to interact with p3a in yeast. Because p3a is primarily localized in the cell membrane and nucleus, and because p3a is a transmembrane protein, we were curious about the function of p3a as a membrane protein in the nucleus. Therefore, among these 9 proteins, we selected the transcription factors TaTRAB1 and TaDOF, which mainly function in the nucleus, for further verification of interaction. BiFC results showed that full length TaTRAB1 did not interact with p3a, while TaDOF and p3a interacted in the nucleus. Therefore, we chose to further study the function of TaDOF in the process of BYDV infection. Those experiments showed that BYDV p3a promoted the expression of *TaHSP70* by inhibiting the function of the TaDOF protein, thus promoting BYDV infection.

DOF transcription factors act as transcriptional activators or repressors involved in a variety of plant-specific biological processes, such as seed maturation and germination, plant hormone and light-mediated regulation, and the plant response to biological and abiotic stresses [10,11]. Previous studies have shown that DOF plays an active role in plant disease resistance by regulating the salicylic acid (SA) and jasmonic acid (JA) signaling pathways in host plants. For example, cucumber plants infected with watermelon mosaic virus and the fungus *Pseudoperonospora cubensis* exhibited upregulated expression of *DOF* gene [40]. Overexpression of the tomato *DOF* gene in tomato enhanced resistance to *Phytophthora oomycetes* [41]. However, there are few studies on the function and mechanism of action of the *DOF* gene in the interaction between viruses and host plants. In this study, the interaction between TaDOF and p3a, which is encoded by BYDV, was investigated to clarify the function of DOF in plant antiviral processes.

In plants, the main biological function of HSP70 is to maintain protein homeostasis and help plants cope with various adverse environmental conditions, such as heat, cold, and drought [42–44]. HSP70 also plays an important role in plant growth and development and is regulated by environmental factors such as light, heat and pathology [44,45]. The knockdown of *HSP70* in wheat affected the wheat growth and development, which was consistent with the results presented in this study [46]. During plant virus infection, HSP70 plays a central role in the formation of membrane-bound replication complexes in certain members of the *Tombusvirus*, *Tobamovirus*, *Potyvirus*, *Dianthovirus*, *Potexvirus*, and *Carmovirus* genera [47]. Previous studies have shown that HSP70 regulates different steps in the assembly of the red clover necrotic mosaic virus replicase complex [48]. The interactions between tomato yellow leaf curl virus and HSP70 in plants and vectors are necessity for viral infection to proceed [49]. In the infected host cell, HSP70 participates in the translocation of CP from the cytoplasm into the nucleus [49]. Studies have also demonstrated that HSP70 influences viral accumulation by mediating the degradation of CP of potato virus A [50]. These studies have focused mainly on the direct interaction of viral proteins with HSP70, wherein HSP70 is hijacked to promote viral infection [51]. This study revealed that TaHSP70 can promote BYDV infection and that p3a does not directly interact with TaHSP70 but promotes the expression of TaHSP70 by inhibiting TaDOF.

**Fig 6:**
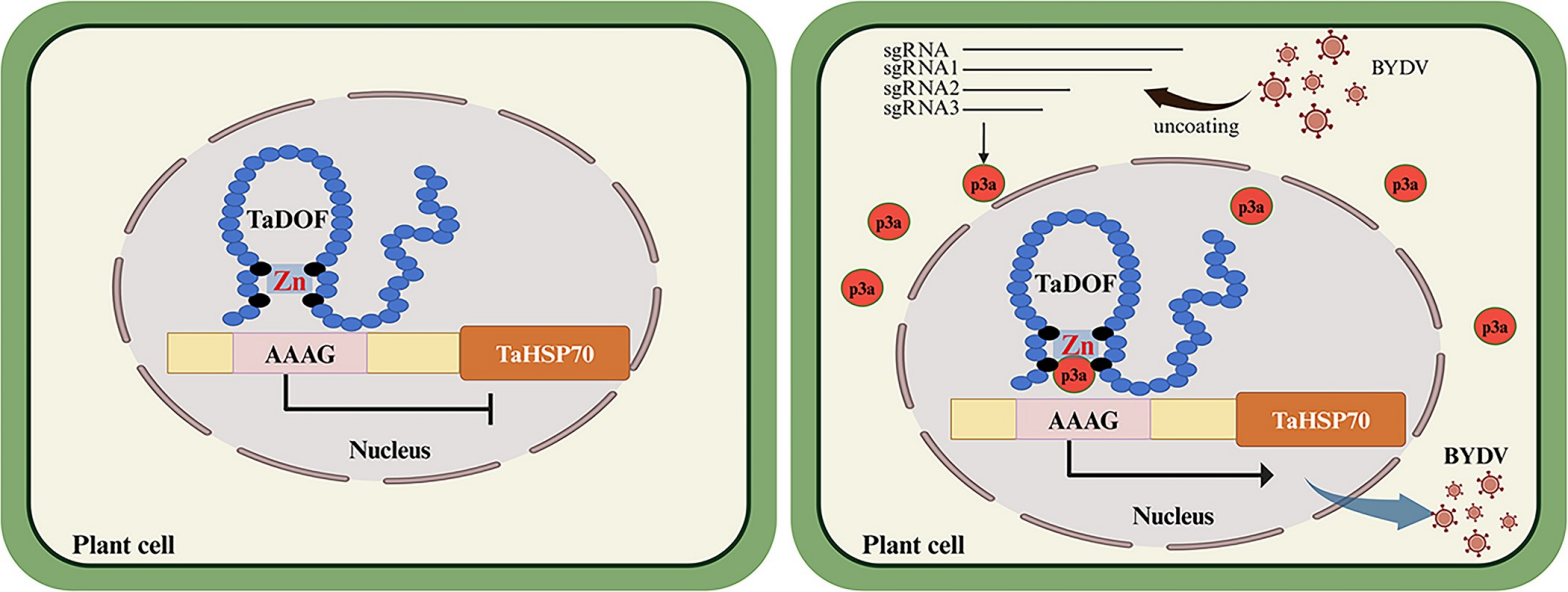
A proposed model illustrating how p3a promotes BYDV infection. p3a weakens the binding ability of the TaDOF zinc finger domain to the *TaHSP70* promoter through interaction with the zinc finger domain of TaDOF, thus alleviating the inhibitory effect of TaDOF on the *TaHSP70* promoter, promoting *TaHSP70* expression, and ultimately promoting BYDV accumulation.

In this study, we investigated the function of the p3a protein encoded by BYDV and found that overexpression of p3a promoted BYDV infection. Further studies showed that p3a could interact with the zinc finger structure of the wheat transcription factor TaDOF, which inhibited the binding of TaDOF to the promoter of the downstream susceptibility gene *TaHSP70*, promoted the expression of *TaHSP70*, and ultimately promoted BYDV infection (Fig 6).

## Materials and methods

### Plant material and virus inoculation

*TaHSP70* knockout ‘CB037’ wheat mutants were provided by Prof. Mingming Xin (China Agricultural University). The methods used to generate the *TaHSP70* knockout plants were reported by Wen et al. [46]. *N*. *benthamiana* plants and wheat plants (‘Fielder’, ‘CB037’ and ‘Xiaoyan6’) were grown in an insect-proof net room under a 16-h light/8-h dark cycle at 24°C. BYDV and aphids were cultured by our laboratory. Five aphids were used to inoculate wheat plants at the two-leaf one-heart stage for BYDV transmission and then killed manually 3 days after inoculation.

### RNA extraction and RT–qPCR analysis

TRIzol reagent (Invitrogen, Carlsbad, CA, USA) was used to extract total RNA from wheat and *N*. *benthamiana* leaves. First-strand cDNA synthesis was performed using a SumOnetube RT Mixture III (gDNA removal) Kit (Summerbio, Shijiazhuang, China) according to the manufacturer’s instructions. A Two×Fast qPCR Master Mixture Kit (DiNing, Beijing, China) and a LightCycler 480 real-time system (Roche, Basel, Switzerland) were used for RT-qPCR.

The elongation factor gene (Gene ID: 543386) was used as an internal reference [50], and the relative expression of the gene was analyzed by the 2^-△△Ct^ method [52]. All the experiments were repeated at least 3 times. The RT–qPCR data were analyzed using IBM SPSS Statistics 29.0 [53]. All primers used in this study are listed in S2 Table. All data used for RT–qPCR are listed in S3 Table.

### Virus detection

RNA extraction and reverse transcription were performed according to the above methods. Rapid Taq Master Mix (Vazyme, Nanjing, China) was used for PCR, and agarose electrophoresis was used to isolate the DNA bands.

### Plasmid construction

*TaDOF* (GenBank accession number XM_044547862.1; Chromosome 6A: 473,574,178–473,576,966) was individually amplified from wheat ‘Xiaoyan6’. The cDNAs of *p3a*, *p3a* mutants (p3a^31-47aa^, p3a^1-7,31-47aa^, p3a^V33G^, p3a^Δ34-37aa^), *TaHSP70* and *TaDOF* were cloned and inserted into the vector pBin-3HA for transient overexpression in *N*. *benthamiana* plants. The cDNA of *p3a* and its mutants (p3a^31-47aa^, p3a^1-7,31-47aa^, p3a^V33G^, p3a^Δ34-37aa^) was cloned and inserted into the pBin-GFP vector for transient overexpression in *N*. *benthamiana* plants. The cDNA of *TaDOF* was cloned and inserted into the pBin-mCherry vector for transient overexpression in *N*. *benthamiana* plants. The cDNAs of *TaDOF* and its mutant TaDOF^△Zn^ were cloned by homologous recombination into the pBin-cYFP vector, and the cDNAs of *p3a* and its mutants (p3a^31-47aa^, p3a^1-7,31-47aa^, p3a^V33G^, p3a^Δ34-37aa^) were cloned and inserted into the pBin-nYFP vector. *Agrobacterium* GV3101 was used for transformation in the BiFC assay [54,55]. The *p3a* and *TaHSP70* genes were cloned and inserted into the pCUB vector for the wheat transgenic experiment. *TaDOF* cDNA was cloned and inserted into the PV101 vector for the systematic overexpression of *TaDOF* in wheat. Partial *TaDOF* cDNA was cloned and inserted into the BSMV-γ vector for VIGS in the wheat cultivar ‘Fielder’. The cDNA of *p3a* was cloned and inserted into the pBT3-N-p3a, pBT3-SUC-p3a, pBT3-STE-p3a and pBT3-N-p3a^V33G^ vectors for the MbY2H assay. The cDNA of *TaDOF* was cloned and inserted into pPR3-N-*TaDOF* for the MbY2H assay. The cDNAs of *TaDOF* and *p3a* were cloned and inserted into pGADT7 for Y1H. The promoter of *TaHSP70* was cloned and inserted into pAbAi for the Y1H assay. The promoter of *TaHSP70* was cloned and inserted into pGreen II-LUC for the dual-luciferase reporter assay.

### *Agrobacterium*-mediated transient expression

*A*. *tumefaciens* strain GV3101 [56] was transformed with the abovementioned plasmids for transient expression. *Agrobacterium* strains harboring different plasmids were cultured in LB medium supplemented with 25 mg/L rifampicin and 50 mg/L kanamycin. After the bacterial culture appeared turbid, the cells were collected by centrifugation and resuspended in buffer containing 10 mmol/L MES, 10 mmol/L MgCl_2_, and 200 μmol/L acetosyringone to an OD_600_ of 0.8. The suspensions were injected into the backs of 4-week-old *N*. *benthamiana* leaves with a 1 mL syringe.

### DAB staining

With reference to the method reported by Daudi et al. [57], changes in H_2_O_2_ in plant leaves were observed via DAB staining. Briefly, plant leaves were immersed in DAB solution (1 mg/mL, pH 3.8), kept at room temperature for 15 min under vacuum and then left at room temperature for 16 h. Next, the leaves were treated with decolorization solution (ethanol:glacial acetic acid:glycerol = 3:1:1) for 3 days, after which the results were photographed and recorded.

### Trypan blue staining

Trypan blue staining was performed as previously described by Zhao et al. [58]. Plant leaves were immersed in trypan blue staining solution (60 mL of ethanol, 20 g of phenol, 20 mL of H_2_O, 20 mL of glycerol, 20 mL of lactic acid, and 20 mg of trypan blue), boiled in a water bath for 10 min, and then incubated for 12 h at room temperature. The samples were destained in saturated chloral hydrate solution (2.5 g of chloral hydrate per 1 mL of water) for 2 days and then equilibrated with 50% (v/v) glycerol for imaging.

### Construction of transgenic wheat lines

According to the method reported by Ishida et al. [59], wheat transgenic experiments were carried out via the *Agrobacterium*-mediated method. Transgenic experiments were carried out with ‘Fielder’ varieties of wheat and the pCUB vector, with the ubi promoter (the vector construction method is described in the “Plasmid construction” section). After *Agrobacterium* infection, possible transgenic wheat plants were obtained through antibiotic screening. After the wheat plants had developed roots, they were transplanted into pots, and PCR detection of the *bar* gene and target gene was performed when the plants had grown to the three-leaf stage. RNA and proteins were extracted from T1, T2 and T3 transgenic wheat leaves for RT-PCR and western blotting analysis. T3 transgenic lines with high *p3a* expression and T2 transgenic lines with high *TaHSP70* expression were selected for subsequent experiments.

### Protein extraction and western blotting

Protein extraction was performed according to the method described by Bai et al. [60]. Briefly, 1 g of plant leaves was collected, placed into a 2 mL tube containing metal beads, mixed with liquid nitrogen and ground with a Tissuelyser (Jingxin, Shanghai, China) at 60 Hz for 90 s. Then, 500 μL of RIPA lysis buffer (Beyotime, Beijing, China) was added to the ground powder. Then, the mixture was shaken thoroughly and left on ice for 30 min. After centrifugation at 13,000×g for 15 min, protein quantification was performed using an enhanced BCA protein assay kit (Beyotime, Beijing, China). Two hundred microliters of the sample was then mixed with 50 μL of 5 × SDS loading buffer and denatured at 100°C for 10 min for acrylamide gel electrophoresis. Eighty micrograms of protein was used for western blotting, and different antibodies, including anti-GFP (ABclonal, Wuhan, China), anti-HA (CST, MA, USA), anti-BYDV [61] (a gift from Jianxiang Wu, Zhejiang University) and anti-Flag (ABclonal, Wuhan, China), were used to detect the protein after it was transferred to nitrocellulose membranes. HRP (horseradish peroxidase), goat anti-mouse IgG (H+L) (ABclonal, Wuhan, China) and HRP-conjugated goat anti-rabbit IgG (H+L) (ABclonal, Wuhan, China) were used as secondary antibodies. The samples were imaged under an ultrasensitive multifunctional imager (UVItec, Cambridge, UK) after incubation with the eECL western blot kit (CWBIO, Beijing, China). Grayscale analysis was performed using ImageJ software, and the actin protein served as the quantitative reference in this analysis.

### BiFC assays

*A*. *tumefaciens* GV3101 strains harboring *p3a* (and mutant)-nYFP and *TaDOF* (and mutant)-cYFP were mixed at a ratio of 1:1 and subsequently infiltrated into 4-week-old *N*. *benthamiana* leaves [32]. Two days after infiltration, fluorescence signals were observed by an Olympus microscope (FV3000 Laser Scanning Confocal Microscope, Tokyo, Japan) using the following excitation wavelengths: GFP, 488 nm; mCherry, 561 nm; and chloroplast autofluorescence, 650 nm. The corresponding emission wavelengths were as follows: GFP, 497–500 nm; mCherry, 585–615 nm; and chloroplast autofluorescence, 650–750 nm.

### Co-IP assay

Co-IP was performed according to the method described by Bai et al. [60]. 3HA-*TaDOF* was coexpressed with GFP-p3a or GFP in *N*. *benthamiana* leaves by the *Agrobacterium*-mediated transient expression method. After 48 h, the leaves (overexpressing *GFP*+3*HA*-*TaDOF*, *GFP-p3a*+3*HA*-*TaDOF* or *GFP-p3a+3HA-TaDOF*^ΔZn^) were collected for protein extraction. Then, 20 μL of anti-GFP Affinity Beads 4FF (Smart-Lifesciences, Changzhou, China) was added, and the samples were washed with balancing/cleaning buffer. The supernatant was removed after centrifugation.

After the beads and proteins were incubated at 4°C for 12 h, the beads were washed again with balance/detergent buffer 6 times. Then, 20 μL of 2 × SDS loading buffer was added, and the mixture was boiled for 10 min to denature the proteins. Western blotting was performed using anti-GFP and anti-HA antibodies.

### Screening of proteins that interact with p3a via the split-ubiquitin Y2H assay

The wheat cDNA library was purchased from Wuhan Protein Interaction Bioengineering Co., Ltd. (Wuhan, China). pBT3-N-p3a, pBT3-SUC-p3a and pBT3-STE-p3a were generated by inserting *p3a* into three decoy vectors. These three plasmids were subsequently transformed into NMY51 yeast cells together with the pOst1-NubI plasmid, which were then cultured in SD/-Trp-Leu solid medium at 30°C for 3–5 days and subsequently transferred to SD/-Trp-Leu-His-Ade yeast medium for approximately 3 days. The bait vector with the best growth was selected for the next library screening. The library screening method was carried out according to the instructions of the DUAL membrane starter Kit (Dualsystems Biotech AG, Zurich, Switzerland).

### Foxtail mosaic virus (FoMV)-mediated gene expression

FoMV-mediated gene expression was performed according to the method described by Jiao et al. [62]. FoMV (PV101) vector was infected into *N*. *benthamiana* leaves via *Agrobacterium*. After 3 days, 0.3 g *N*. *benthamiana* leaves was taken and ground into a powder in liquid nitrogen. The powder was dissolved into a homogenate with 10% (w/v) 0.01 mol/L phosphate buffer and used for rub inoculation onto both leaves of two-leaf-stage wheat seedlings (‘Xiaoyan 6’). After inoculation, wheat leaves were evenly sprayed with enzyme-free sterile water, bagged, and placed under high humidity without light for 24 h. They were then transferred to normal culture conditions. FoMV containing TaDOF was the experimental group, and FoMV containing GFP was the control group. Western blotting was performed 15 days after FoMV inoculation to determine whether TaDOF was expressed.

### BSMV-mediated gene silencing

The BSMV-γ-*TaDOF* vector was constructed, with γ0 serving as the negative control. *In vitro* transcription was conducted with 1 μg of construct and a RiboMAX Large Scale RNA Production System (Promega, St. Louis, MO, USA) according to the manufacturer’s instructions. The wheat cultivar ‘Fielder’ was mechanically inoculated at the two-leaf stage with a mixture of 100 ng of BSMV-α, BSMV-β and BSMV-γ at a ratio of 1:1:1 in 200 μL of buffer [60,63] (1 mol/L sodium phosphate, pH 7.0, to a final concentration of 42.9 mmol/L; 0.28–0.56 mol/L bentonite). After inoculation, the wheat plants were sprayed with water, after which the plants were incubated at 24°C in the dark for 12 h. Then, cultivation under light was performed. After 2 weeks, the leaves were collected, and the gene silencing efficiency was tested via RT-qPCR.

### Yeast one-hybrid assay

The Y1H assay was performed according to the instructions of the Matchmaker Gold Yeast One-Hybrid Library Screening System (Clontech, Peking, China). First, the AbA concentration was screened (Coolaber, Peking, China). The AbA concentration that inhibited only pAbAi-TaHSP70 promoter self-activation was selected for subsequent interaction verification. AD-TaDOF and AD-p3a were subsequently transferred into Y1H yeast cells harboring the *TaHSP70* promoter. The pGADT7 plasmid was used as a negative control, and the AD-P53 and pAbAi-P53 plasmids were used as positive controls. Colony growth was observed on SD/-Leu+0.27 μmol/L AbA solid medium at 30°C for 3–5 days, after which the interactions were analyzed.

### Dual-luciferase assay

The recombinant pGreenII-TaHSP70 promoter-LUC vector was coexpressed with *p3a* (and its mutants) and/or *TaDOF* in the leaves of *N*. *benthamiana* for 3 days. Then, 15 mg/mL D-luciferin potassium salt (Beyotime, Beijing, China) was added for 10 min, and the fluorescence signal was observed via a multispectral dynamic fluorescence microscope imaging system (Plant View 100, Canton, China).

## Supporting information

S1 Fig Phenotype and identification of wheat plants overexpressing *p3a* (related to Fig 1). (a) Symptoms of *p3a*-overexpressing (*p3a*-OE) and wild-type (WT) ‘Fielder’ wheat plants inoculated with BYDV. (b-c) Reverse transcription PCR (RT–PCR; b) and western blotting (c) were conducted to validate the transgenic wheat *p3a*-OE plants. The WT plants were used as a negative control. Elongation factor (EF) was used as an internal reference.
(TIF)

S2 Fig Protein secondary structure analysis and subcellular localization. (**a**) TMHMM was used to predict the transmembrane structure of p3a. (**b**) Subcellular localization of GFP-p3a in *Nicotiana benthamiana* cells. (**c**) TaDOF was predicted by NCBI Conserved Domain Search to contain a zinc finger domain. Zf-DOF is the zinc finger domain.
(TIF)

S3 Fig Aureobasidin A (AbA) concentration screening for inhibition of pAbAi-TaHSP70 promoter self-activation. (TIF)

S4 Fig Phenotype and schematic diagram of the strategy used to generate *TaHSP70* knockout mutants (related to Fig 4). (**a**) The sgRNA sequence for *TaHSP70* (underlined) targets a conserved region in the second exon of *TaHSP70*. The protospacer-adjacent motif (PAM) sequence is highlighted in red. Sequencing results from the two mutant lines, KO-55 and KO-31, indicate base insertion (+) and deletion (–) with respect to the wild-type sequences of different *TaHSP70* homeologs. Blue letters and dashes indicate insertions and deletions, respectively. Numbers in parentheses represent the knockout gap lengths of the mutants. (**b**) Knockout *TaHSP70* inhibits the formation of wheat yellowing symptoms caused by BYDV.
(TIF)

S5 Fig Phenotype and identification of wheat plants overexpressing *TaHSP70* (related to Fig 4). (**a**) *TaHSP70*-overexpressing (TaHSP70-OE) and wild-type (WT) ‘Fielder’ wheat plants. (**b**) Symptoms of TaHSP70-OE and WT ‘Fielder’ wheat plants inoculated with BYDV. (**c and d**) Quantitative reverse transcription PCR (RT–qPCR; c) and western blotting (d) were conducted to validate the transgenic wheat TaHSP70-OE line. WT plants were used as a negative control.
(TIF)

S6 Fig Colocalization analysis of the GFP-p3a mutant and mCherry-TaDOF in *Nicotiana benthamiana* cells. DAPI was used to stain the nucleus.
(TIF)

S1 Table List of the identified p3a-interacting proteins. (XLSX)

S2 Table All primers used in this study. (XLSX)

S3 Table Data for statistical analysis. (XLSX)
