# Supplementary figures and images for: A viral p3a protein targets and inhibits TaDOF transcription factors to promote the expression of susceptibility genes and facilitate viral infection
Source: PLoS Pathog. 2024 Nov 7;20(11):e1012680. doi: 10.1371/journal.ppat.1012680 (PMC11542804; doi:10.1371/journal.ppat.1012680)

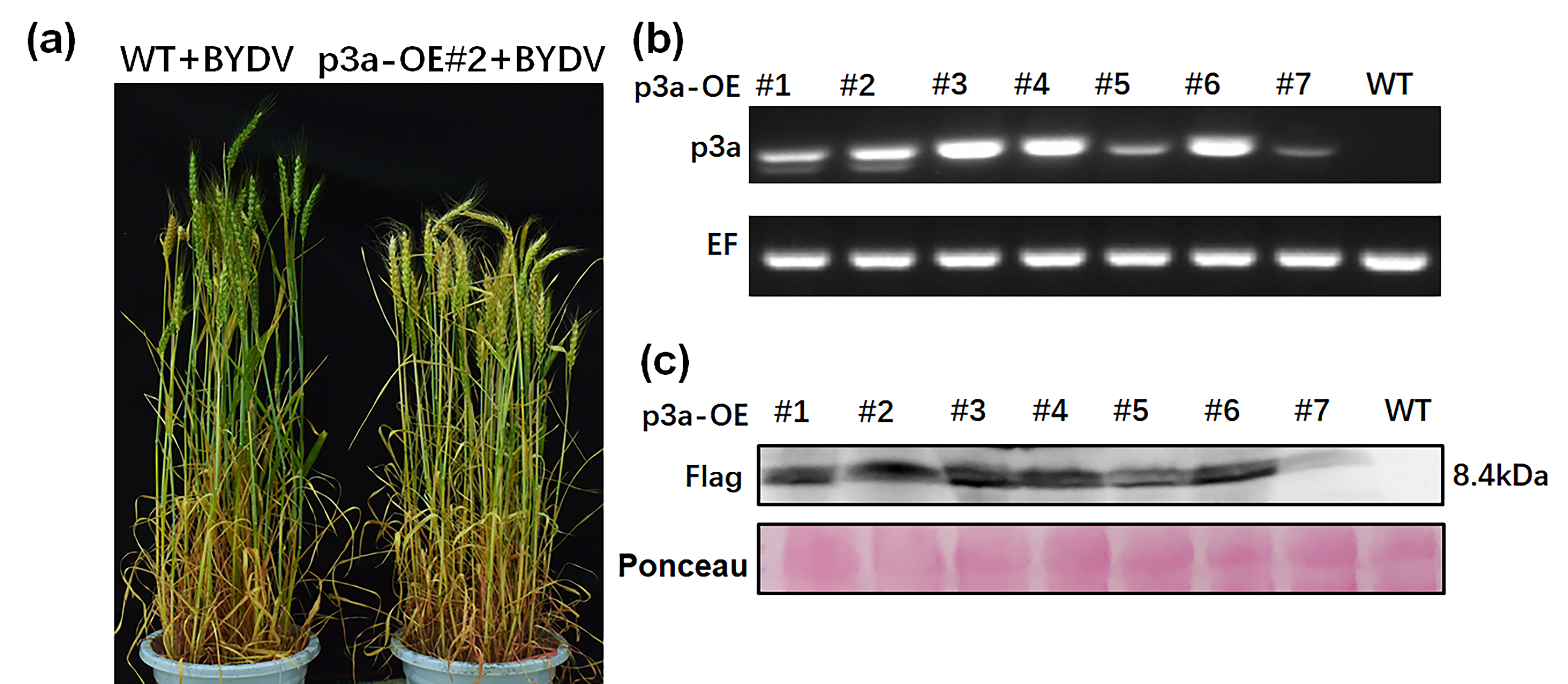

Supplement: S1 Fig — (a) Symptoms of p3a-overexpressing (p3a-OE) and wild-type (WT) ‘Fielder’ wheat plants inoculated with BYDV. (b-c) Reverse transcription PCR (RT–PCR; b) and western blotting (c) were conducted to validate the transgenic wheat p3a-OE plants. The WT plants were used as a negative control. Elongation factor (EF) was used as an internal reference. (TIF) [file ppat.1012680.s001.tif]

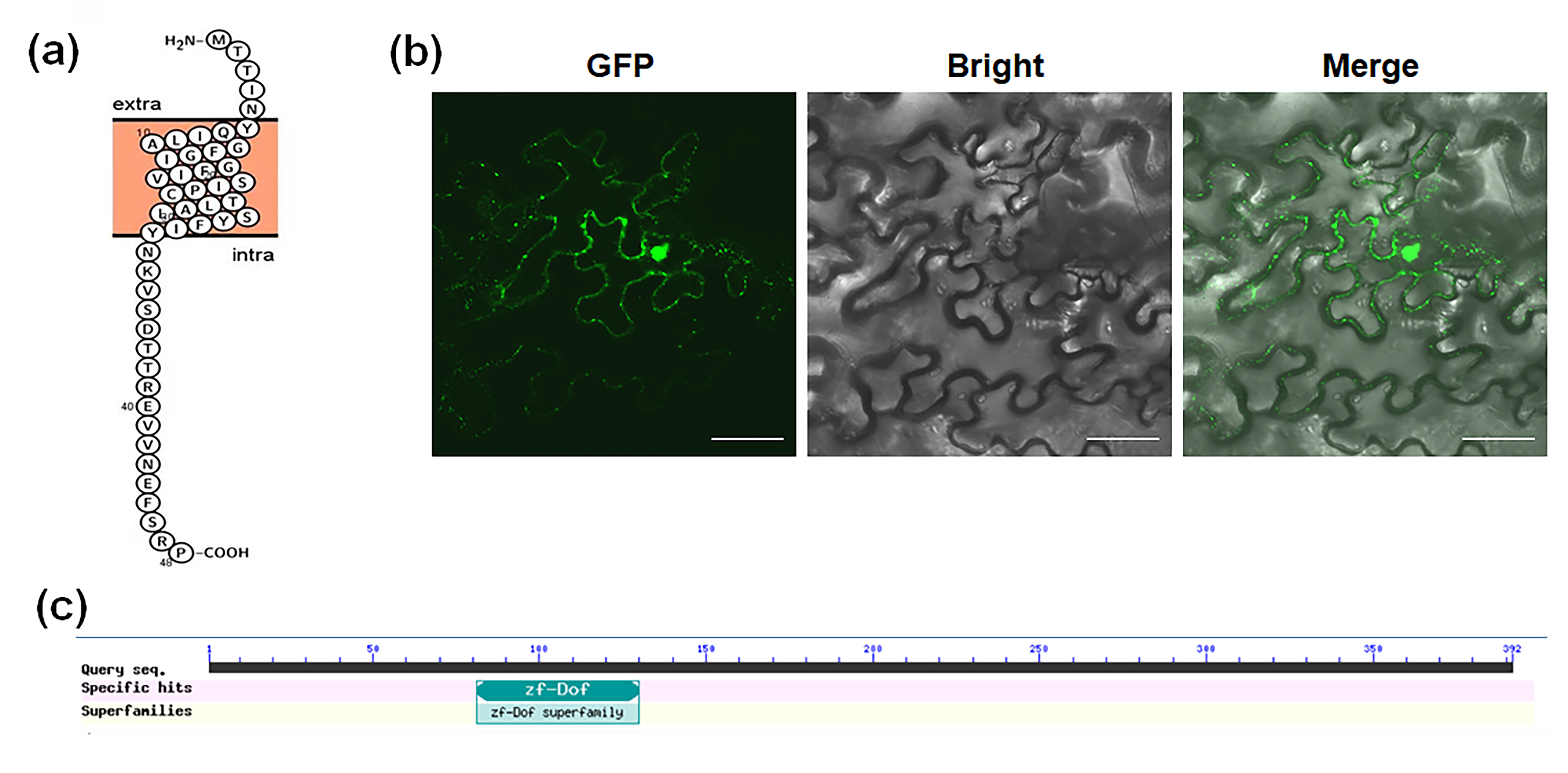

Supplement: S2 Fig — (a) TMHMM was used to predict the transmembrane structure of p3a. (b) Subcellular localization of GFP-p3a in Nicotiana benthamiana cells. (c) TaDOF was predicted by NCBI Conserved Domain Search to contain a zinc finger domain. Zf-DOF is the zinc finger domain. (TIF) [file ppat.1012680.s002.tif]

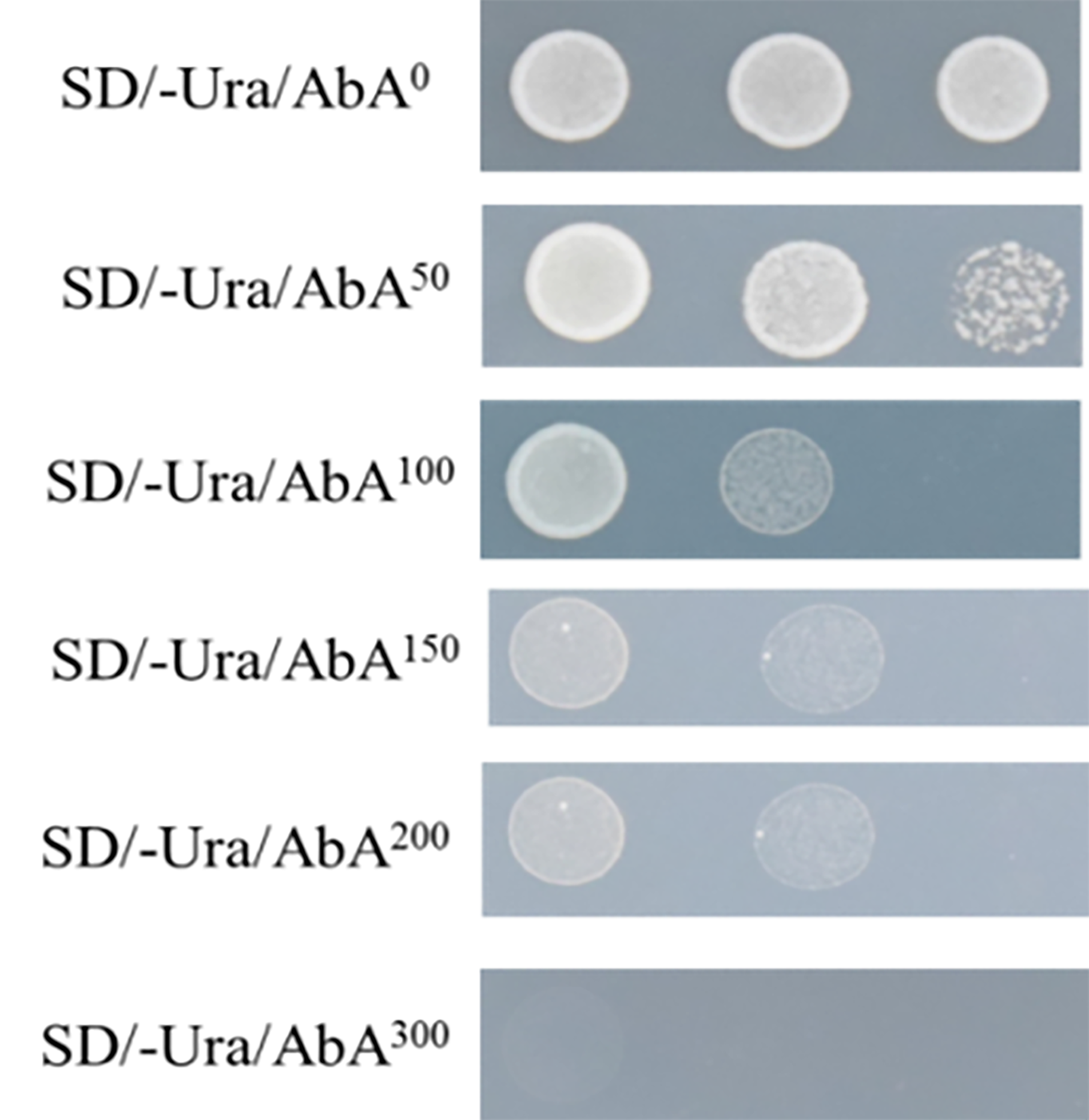

Supplement: S3 Fig — (TIF) [file ppat.1012680.s003.tif]

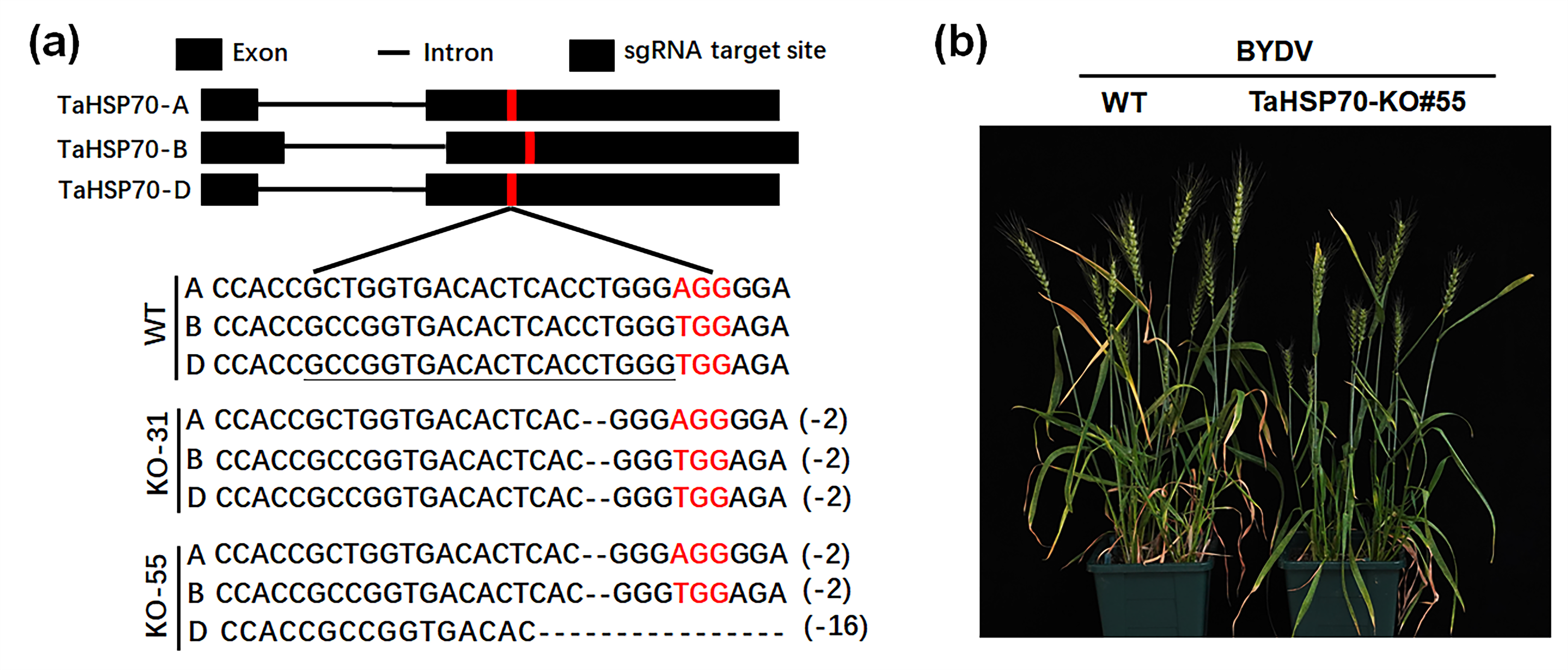

Supplement: S4 Fig — (a) The sgRNA sequence for TaHSP70 (underlined) targets a conserved region in the second exon of TaHSP70. The protospacer-adjacent motif (PAM) sequence is highlighted in red. Sequencing results from the two mutant lines, KO-55 and KO-31, indicate base insertion (+) and deletion (–) with respect to the wild-type sequences of different TaHSP70 homeologs. Blue letters and dashes indicate insertions and deletions, respectively. Numbers in parentheses represent the knockout gap lengths of the mutants. (b) Knockout TaHSP70 inhibits the formation of wheat yellowing symptoms caused by BYDV. (TIF) [file ppat.1012680.s004.tif]

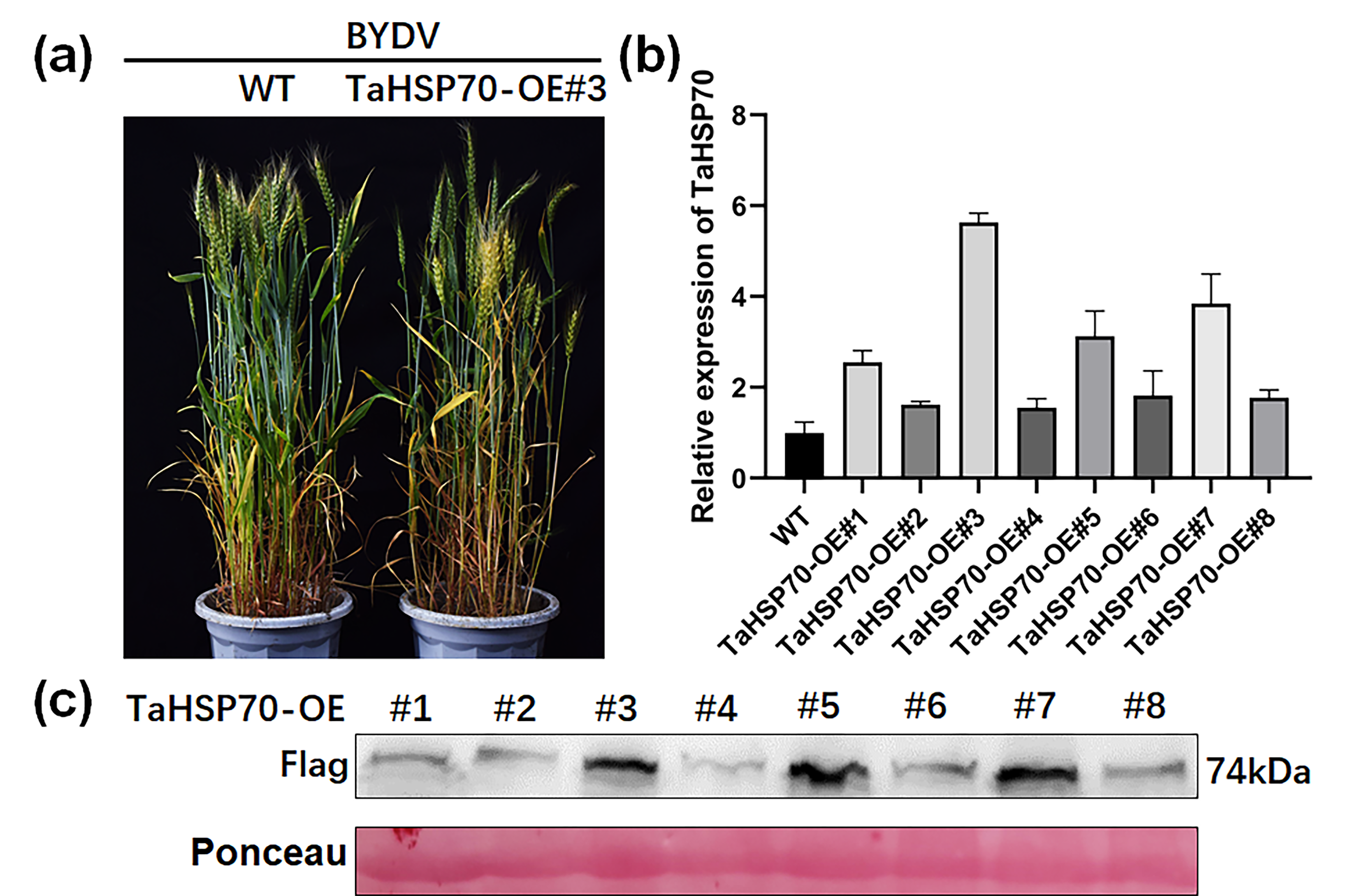

Supplement: S5 Fig — (a) TaHSP70-overexpressing (TaHSP70-OE) and wild-type (WT) ‘Fielder’ wheat plants. (b) Symptoms of TaHSP70-OE and WT ‘Fielder’ wheat plants inoculated with BYDV. (c and d) Quantitative reverse transcription PCR (RT–qPCR; c) and western blotting (d) were conducted to validate the transgenic wheat TaHSP70-OE line. WT plants were used as a negative control. (TIF) [file ppat.1012680.s005.tif]

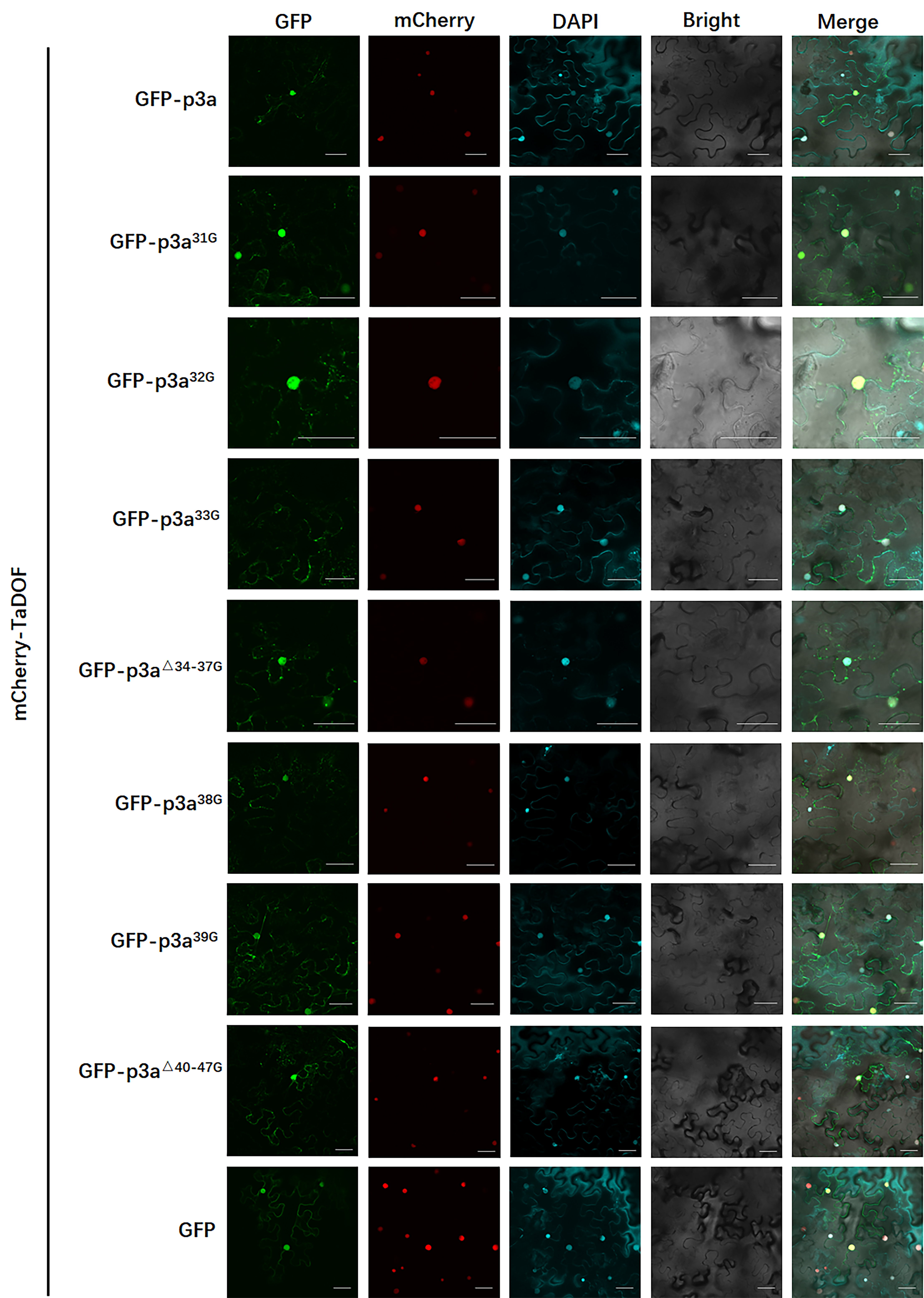

Supplement: S6 Fig — DAPI was used to stain the nucleus. (TIF) [file ppat.1012680.s006.tif]
